# Usability of Videogame-Based Dexterity Training in the Early Rehabilitation Phase of Stroke Patients: A Pilot Study

**DOI:** 10.3389/fneur.2017.00654

**Published:** 2017-12-08

**Authors:** Tim Vanbellingen, Suzanne J. Filius, Thomas Nyffeler, Erwin E. H. van Wegen

**Affiliations:** ^1^Neurology and Neurorehabilitation Center, Luzerner Kantonsspital, Luzern, Switzerland; ^2^Gerontechnology and Rehabilitation Group, University of Bern, Bern, Switzerland; ^3^Faculty of Behavioral and Movement Sciences, Amsterdam Movement Sciences, VU University Amsterdam, Amsterdam, Netherlands; ^4^Mechanical, Marine and Materials Engineering, Technical University of Delft, Delft, Netherlands; ^5^Department of Rehabilitation Medicine, Amsterdam Movement Sciences, Amsterdam Neurosciences, Vrije Universiteit Medical Center, Amsterdam, Netherlands

**Keywords:** stroke, dexterity, videogame-based training, Leap Motion Controller, virtual reality, usability

## Abstract

**Background:**

Approximately 70–80% of stroke survivors have limited activities of daily living, mainly due to dexterous problems. Videogame-based training (VBT) along with virtual reality seems to be beneficial to train upper limb function.

**Objective:**

To evaluate the usability of VBT using the Leap Motion Controller (LMC) to train fine manual dexterity in the early rehabilitation phase of stroke patients as an add-on to conventional therapy. Additionally, this study aimed to estimate the feasibility and potential efficacy of the VBT.

**Methods:**

During 3 months, 64 stroke patients were screened for eligibility, 13 stroke patients were included (4 women and 9 men; age range: 24–91 years; mean time post stroke: 28.2 days).

**Intervention:**

Nine sessions of 30 min VBT, three times per week as an add-on to conventional therapy with stroke inpatients.

**Outcome measures:**

Primary outcome was the usability of the system measured with the System Usability Scale. Secondary outcomes concerning feasibility were the compliance rate calculated from the total time spent on the intervention (TT) compared to planned time, the opinion of participants via open-end questions, and the level of active participation measured with the Pittsburgh Rehabilitation Participation Scale. Regarding the potential efficacy secondary outcomes were: functional dexterity measured with the Nine Hole Peg Test (NHPT), subjective dexterity measured with the Dexterity Questionnaire 24, grip strength measured with the Jamar dynamometer, and motor impairment of the upper limb measured with the Fugl-Meyer Upper Extremity (FM-UE) scale.

**Results:**

Primarily, the usability of the system was good to excellent. The patient’s perception of usability remained stable over a mean period of 3 weeks of VBT. Secondly, the compliance rate was good, and the level of active participation varied between good and very good. The opinion of the participants revealed that despite individual differences, the overall impression of the therapy and device was good. Patients showed significant improvements in hand dexterity. No changes were found in motor impairment of the upper limb (FM-UE) during intervention.

**Conclusion:**

VBT using LMC is a usable rehabilitation tool to train dexterity in the early rehabilitation phase of stroke inpatients.

## Introduction

Stroke is a serious global health-care problem and is one of the greatest causes of acquired adult disability (1). Approximately 70–80% of stroke survivors have limited activities of daily living after discharge home (2). They experience for example difficulties with feeding, dressing, and grooming, mainly due to impaired dexterity (3, 4). Neurorehabilitation plays a major role in the treatment of stroke patients (1), in which improving dexterity is a core element of treatment protocols (5).

Most improvements in upper limb function usually occur within the first month poststroke (6–8). It is suggested that neurorehabilitation can enhance neurological recovery (9, 10) and to elicit neuro-plastic adaptations it is important that exercise programs are intensive, highly repetitive, and task-specific (5, 11–13). Additionally, it is recommended to start interventions early poststroke because of heightened brain plasticity in this period (7, 9).

To further enhance upper limb outcome, research continues to investigate new approaches (14). An upcoming therapeutic method is videogame-based training (VBT) along with virtual reality (VR) (15, 16). Recent meta-analyses claim that there is moderate evidence that VR training may be beneficial for upper limb recovery after stroke (3, 17). VBT has several advantages such as variety in games and variance in artificial environments or stimuli and it can be used in a home-based situation (12, 15). These advantages could improve the motivation to sustain a repetitive intervention. Another important property of VBT is online feedback, which can increase the effectiveness of motor learning-based training by perceiving and correcting movement error (18, 19). Especially in VBT, visual and auditory feedback is important because there is no sensory feedback of real-world object in the hands.

Devices such as Microsoft Kinect™ and Nintendo Wii-Fit could be used in VBT [e.g., (20, 21)]. However, these devices fail to detect fine hand and finger movements (15), which is needed to train dexterity. Several studies report moderate improvements in dexterity using VBT in stroke, but these systems are not commercially available (22–25). A commercially available device called Leap Motion Controller (LMC) is a low-cost, low-complexity optoelectronic system, which can track hand movements (15, 26). The LMC is delivered with software in which several videogames can be uploaded. So far, one explorative small sample feasibility study looked at the use of LMC in four chronic stroke patients and demonstrated good compliance but failed to show significant effects on hand dexterity (15). However, while important, the level of active participation is not sufficient as feasibility measure alone. Active participation can be influenced by several factors such as intrinsic motivation to recover, enthusiasm of the therapist, system usability, and complexity etcetera. Therefore, the present study evaluates the usability of the LMC system with the System Usability Scale (SUS) (27), which is well validated to evaluate new technologies, including software. In addition to system usability, this pilot study aimed to get a comprehensive estimation of the feasibility of the VBT. Consequently, the compliance rate, the level of active participation, and the opinion of the participants were systematically evaluated. Furthermore, we aimed to evaluate the potential efficacy of a specific dexterity LMC training program by evaluating recovery of motor function of the arm, grip strength, and dexterity. We hypothesized that VBT using the LMC, initiated within the early rehabilitation phase (5), would be a usable tool to train manual dexterity in stroke patients as an add-on therapy. Moreover, we expected the compliance rate and level of active participation to be good. Concerning efficacy, we hypothesized that VBT with LMC improves hand dexterity.

## Materials and Methods

The study was approved by the Ethikkommission Nordwest- und Zentralschweiz of the canton Lucerne. All patients gave written informed consent according to the latest declaration of Helsinki (2013).

### Participants

The participants were recruited through medical chart review and regular visits between April and May 2017. Each patient in this study received standard neurorehabilitation care during their hospitalization. Patients were selected according to the following criteria: inclusion criteria were: (1) written informed consent, (2) aged above 18 years, (3) a first-ever stroke in the past 24 h until 3 months, reflecting the early rehabilitation phase (5) (4) experience upper limb impairments due to stroke (Nine Hole Peg Test > 19 s with at least one side) (28), (5) at least able to perform ante-flexion with their upper arm and extent one or more fingers against gravity (3 ≤ Medical Research Council scale < 5) (29), and (6) the participants had to be able to understand the instructions and assessments in German. Exclusion criteria were: (1) severe cognitive impairments (Montreal Cognitive assessment: MoCa < 14) (30), (2) severe apraxia (Apraxia Screen of TULIA < 9) (31), (3) aphasia (Language screening test < 15) (32), (4) severe self-reported pain, or (5) other severe orthopedic problems of the upper limb impairing participation.

### Procedure

The intervention consisted of nine training sessions of 30 min, spread out over a period of 3 weeks, this means three training sessions per week. An intake session was planned with eligible patients. Upon consent, the baseline measures of dexterity, grip strength, and motor impairment of the upper limb were collected. This was, if possible, followed by the first training and evaluation of the usability of the system. The level of active participation and time spent on the intervention were measured during each training session. The second, fourth, fifth, seventh, and eighth session contained only training. In the third, sixth, and ninth session, dexterity and grip strength were measured followed by the training and evaluation of the usability. The reassessment of the motor impairment of the upper limb and evaluation of the open-end questions were at the ninth session. The present study aimed to follow the procedure schedule as shown in Table 1.

**Table 1:** Schedule of aimed procedure.

Week 1	Day 1	60 min	T0 + T1	NHPT → Jamar → DextQ-24 → FM-UE → Training → SUS (T1)
	Day 2	30 min	T2	Training
	Day 3	45 min	T3	NHPT → Jamar → DextQ-24 → Training → SUS (T3)

Week 2	Day 1	30 min	T4	Training
	Day 2	30 min	T5	Training
	Day 3	45 min	T6	NHPT → Jamar → DextQ-24 → Training → SUS (T6)

Week 3	Day 1	30 min	T7	Training
	Day 2	30 min	T8	Training
	Day 3	60 min	T9	NHPT → Jamar → DextQ-24 → FM-UE → Training → SUS (T9) → Interview

*Interview with open-end questions. The arrows represent the order of actions during the sessions*.

*T, training session; SUS, Systems Usability Scale; NHPT, Nine Hole Peg Test; DextQ-24, Dexterity Questionnaire 24; FM-UE, Upper Extremity motor section of the Fugl-Meyer Assessment, measures motor impairment of the upper limb*.

### Intervention

**Figure 1:**
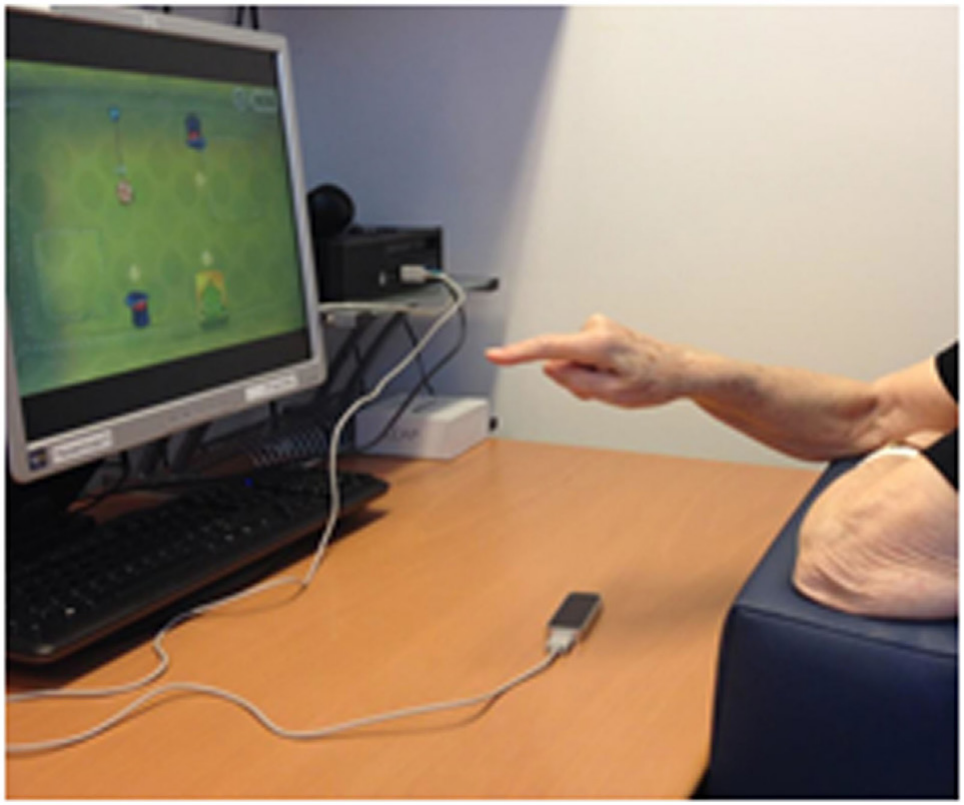
Research setup. Courtesy of S. Filius.

Each training session was performed on a desktop computer at the occupational ward of the neurorehabilitation. The participants (if not in a wheelchair) were sitting on a chair with a rectangular pillow on their lap, so that the elbows could rest on the pillow (see Figure 1).

The LMC was placed on a table in front of the participant between the body and the computer screen. During each session, the principal investigator sat next to the participants, providing (if needed) online feedback via verbal, visual, and/or physical instructions.

The LMC incorporates three infrared emitters and two charge-coupled device cameras for capturing the motion of both hands, wrists, and forearms (15, 26). The light of the infrared emitters reflects back from the surfaces of the hands, so no markers are needed (33). Weichert et al. (34) reported that the LMC has an accuracy of 0.2 and 1.2 mm in a static and dynamic setup, respectively.

**Figure 2:**
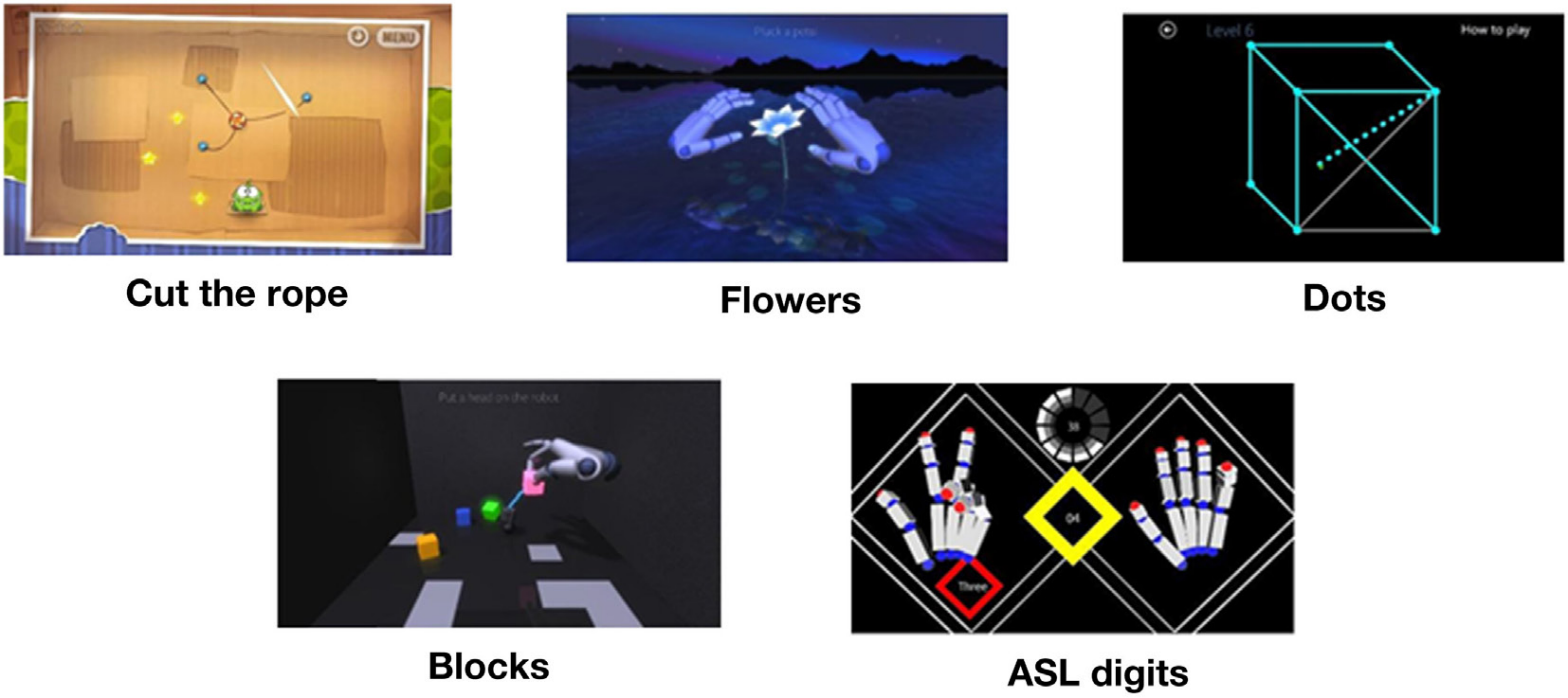
Games. Retrieved from Leap Motion App Store© (https://apps.leapmotion.com/?sign_up=true).

In February and March 2017, all free access games in the Leap Motion App Store© (https://apps.leapmotion.com/?sign_up=true) were evaluated to determine if the games contained key components of dexterity movements: alternating finger, pincer grasp, fine pointing, and palmar extension/flexion. Five games were selected: Dots Trial, Cut the Rope, Playground, and American Sign Language Digits. Playground contains two games named Blocks and Flower. See Figure 2 for an impression of the games.

Each therapy session contained about 6 min per game, and the sequence of the five games was randomized by the function “randperm” using MATLAB R2013b (Mathworks, Natick, MA, USA). Each game is played with both hands starting with the non-affected hand.

A HP EliteDesk 800 (Intel Core i5) desktop computer was used, with a 19-inch computer screen with an aspect ratio 4:3 (1,280 × 768).

### Outcome Measures

All outcome measurements were collected and/or rated by a single trained assessor (SF) to optimize standardization of outcome measurement.

#### Primary Outcome

The primary outcome measure was self-reported system usability, evaluated by the SUS (27). Two usability aspects are important: that the LMC is able to track the impaired hand and that patients cognitively understand the VBT. The SUS is a generalized usability measure which collects users’ subjective perception of interaction with different interfaces (27).

The SUS has ten items with a 5-point scale from 1 (strongly disagree) to 5 (strongly agree), with a range from 0 to 100 (35, 36) and takes into account three usability criteria: effectiveness, efficiency, and satisfaction (36). Brooke stated that the SUS is a robust and reliable evaluation tool with high face validity, but no qualitative values of reliability and validity were found. We used a German translation of the SUS, translated by a native speaker. A system is acceptably usable from a SUS score upwards of 70, with “good products” scoring between high 70s and upper 80s (27). The participants were asked to fill-in the SUS independently.

#### Secondary Outcomes

##### Feasibility

Feasibility was comprehensively measured by (1) the compliance rate which was determined by the ratio of the total time spent on intervention (TT) and planned time (PT), (2) the level of active participation, measured with the Pittsburgh Rehabilitation Participation Scale (PRPS) (37), and (3) the subjective opinion of the participants, evaluated by open-end questions in an interview form.

To determine the compliance rate, the time spent on every training session was recorded by the principal investigator. The TT was compared to the PT (4 h 30 min) to evaluate if patients were able and willing to complete the intervention. Patients who were discharged from hospital before end of intervention were excluded from the compliance analysis.

The level of active participation was measured with the PRPS. The PRPS is a six-point scale from 1 “refusal to participate” to 6 “excellent participation” (15) and is a reliable and valid therapist-rated measure of inpatients participation in occupational therapy (intraclass correlation coefficient: ICC = 0.91) and physical therapy (ICC = 0.96) (37).

The open-end questions were about (1) the overall impression of the therapy/LMC device, (2) the duration of the therapy, (3) the potential home-use, (4) possible improvements of the system, (5) overall remarks or commentary, (6) the quality of the instructions of the therapist, and (7) the most favorite and (8) the least favorite game. The answers were given orally and written down by the principal investigator. See Appendix S1 in Supplementary Material for the open-end questions in German.

##### Efficacy

Since the focus of the training program was dexterity, several standardized outcome measures were performed: (1) dexterity measured with the Nine Hole Peg Test (NHPT), (2) subjective, self-reported dexterity measured with the Dexterity Questionnaire 24 (DextQ-24) (38), (3) grip strength measured with the Jamar dynamometer (39), and (4) the motor impairment of the upper limb measured with the Upper Extremity motor section of the Fugl-Meyer Assessment (FM-UE) (40).

The NHPT is a hand function test, which consists of a plastic peg board (25.0 cm × 12.7 cm × 2.3 cm) with nine holes (2.54 cm between the holes) and nine pegs (3.2 cm long, 0.64 cm wide) (41). Chen et al. found a good test–retest reproducibility of the NHPT (ICC = 0.85 with more-affected side). The participant had to put the nine pegs in the peg board as fast as possible, one at the time with one hand only, and remove them again. The test was performed two times per hand, with the non-affected hand first. The time it takes to fulfill the second trial with the more-affected hand was used for the analysis.

The DextQ-24 is a patient reported outcome measure that evaluates the performance and independence of daily dexterity activities. The DextQ-24 is a valid and reliable (ICC = 0.91; measurement error = 2.9) measurement in patients with Parkinson’s disease (38). The DextQ-24 has 24 items with 12 items uni-manual and 12 items bi-manual. The scale goes from 1 “no problems” to 4 “unable to perform the task and needing aid from a third person” (38).

The grip strength is measured with the Jamar baseline^®^ hydraulic hand dynamometer. The Jamar dynamometer is a reliable tool with a high intra-examiner reliability (ICC = 0.97–0.99) and a SEM between 0.98 and 1.69 kg in chronic stroke patients (39). The grip strength is assessed following the recommendations of American Society of Hand Therapists (ASHT) with the shoulder adducted, elbow flexed at 90°, forearm in neutral position, and the second position of the handle is used (42). The participants have to perform the grip strength test three times alternating per hand, and the averaged value of the affected hand is used for analysis.

The use of FM-UE is worldwide recommended for clinical trials of stroke rehabilitation as an evaluation of recovery in the poststroke hemiplegic patient (43). The FM-UE has 34 items which are scored from 0 to 2, with a total range from 0 to 66 points (43).

### Statistical Analysis

Descriptive statistics were used to calculate the group mean (*M*), SD, and SEM of clinical and demographic variables.

The distributions of the outcome measures (SUS, PRPS, NHPT, DextQ-24, Jamar, and FM-UE) were examined for normality of distribution to select either the parametric one-way repeated measure ANOVA or the non-parametric Friedman’s ANOVA. Wilcoxon Signed Rank Test was used in addition to the non-parametric Friedman’s ANOVA. The compliance rate is calculated by: *compliance rate* = TT/PT × 100%. A compliance rate of 80% was defined as good (44). The level of active participation was evaluated by the principal investigator per game. The PRPS scores per training was the average PRPS score of all the games played in that session. The answers of the open-end questions 1, 2, 3, and 6 were grouped in positive, neutral, and negative answers, so that the answers could be organized and analyzed. For the remaining open-end questions, a descriptive summary of the answers is given.

Statistical analyses were performed using the SPSS statistical software system (IBM SPSS Statistics for Windows, Version 22.0. Armonk, NY, USA: IBM Corp.), and a confidence level of 95% was used, so that level of significance was set at *p* = 0.05, two-tailed.

## Results

### Descriptive Data

During the recruitment period, 64 stroke patients admitted to the neurorehabilitation ward were screened for eligibility. Fifteen patients who were potentially eligible were selected. Two patients did not accept informed consent and were excluded. Thirteen patients started the 3 weeks LMC dexterity training program. Clinical and demographic characteristics are presented in Table 2.

**Table 2:** Clinical and demographic characteristics.

Descriptive data	*N* = 13
Gender, male/female, *n*	9/4
Age, years (range)	68.2 ± 17.5 (24–91)
Time post-stroke at inclusion, days (range)	28.2 ± 23.2 (8–88)
Type of stroke, *n*	
Ischemic CVA, A. cerebri media	5
Ischemic CVA, A. cerebri posterior	7
Hemorrhage, CVA	1
Handedness, *n*	
Right/left	11/2
Paretic side, *n*	
Right	8
Left	4
Both	1
Dominant side affected, *n*	7
NHPT, s	50.0 ± 26.9 (17–60)
DextQ-24	41.4 ± 15.8 (27–70)
Fugl Meyer Arm score	57.5 ± 9.3 (42–65)
MoCA	21.7 ± 5.7 (14–30)

*All data are presented by the mean ± SD or otherwise stated*.

*N or n, number of participants; NHPT, Nine Hole Peg Test; DextQ-24, Dexterity Questionnaire-24; MoCA, Montreal Cognitive Assessment; CVA, cerebrovascular accident*.

Eight of the thirteen patients could complete the whole training program. One participant experienced a new stroke between the seventh and eighth session, and therefore, follow-up measures were excluded from the main analysis. Three other patients were discharged earlier. And finally, one older patient, a 62-year old male, stopped the intervention during the second training session due to lack of motivation. Importantly, there were no severe intervention-related adverse events like severe shoulder pain or severe fatigue.

Overall analyses revealed few missing data in the outcome measures: SUS (3.1%) and DextQ-24 (0.65%). Average imputation was used to correct these gaps. Furthermore, one participant was not able to perform the NHPT with his affected hand and is not included in the NHPT analysis. The other outcome measures had no missing data.

### Outcomes

#### Primary Outcome

**Figure 3:**
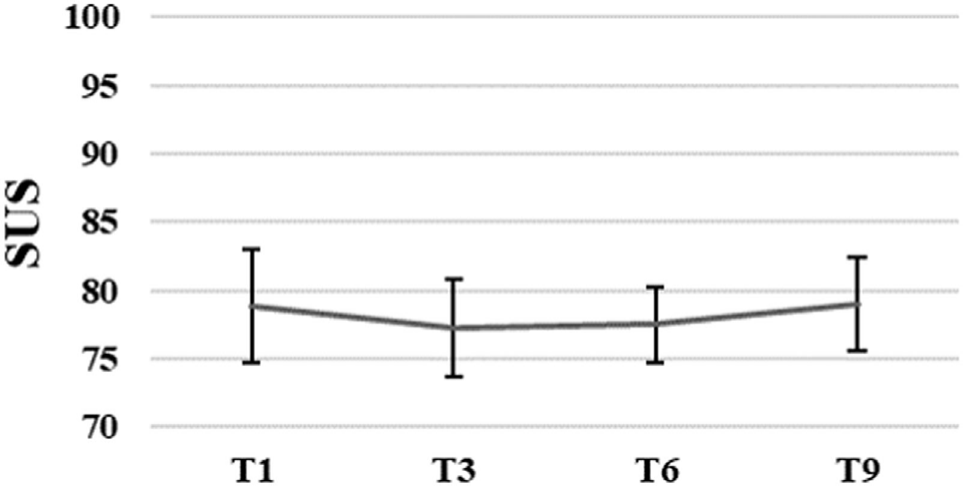
Group means with SEM for the results of the System Usability Scale (SUS) scores measured at different time points.

The average SUS score of all participants (*N* = 13) was 75.4, SD = 13.8 after the first training session. Patients’ perception of usability of the system remained unchanged, *F*_(3, 21)_ = 0.09, *p* = 0.96 over time. The average SUS score of the eight participants who finished the intervention was *M* = 78.9, SD = 11.6 after the first, and *M* = 79.1, SD = 9.7 after the ninth training session (for more details see Figure 3).

#### Secondary Outcomes

##### Feasibility

The average TT spent on the intervention was, *M* = 3 h 56 min, SD = 1 h 16 min with a range of 42 min to 4 h 45 min. The compliance rate of the participants was 87.4%.

**Figure 4:**
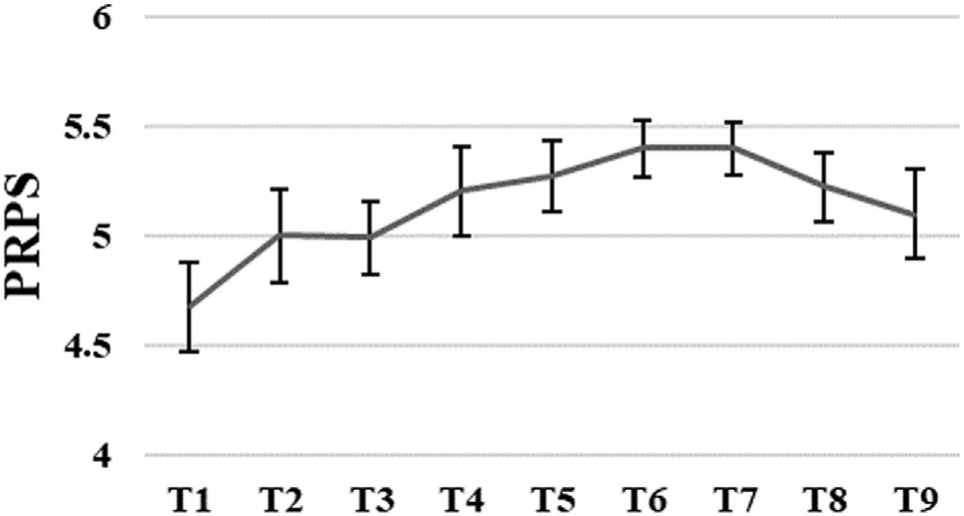
Group means with SEM for the results of the Pittsburgh Rehabilitation Participation Scale (PRPS) scores measured at different time points.

The average level of active participation measured with PRPS varied between good 4.7 and very good 5.4. The PRPS scores slightly increased until sixth training session followed by a small decrease after the seventh training session. However, these changes were not significant, *F*_(1.05, 0.39)_ = 2.71, *p* = 0.07 (for more details, see Figure 4).

##### Efficacy

Nine Hole Peg Test scores decreased significantly during intervention, $\chi_{\left(3\right)}^{2}=15.34$, *p* < 0.00. The NHPT scores significantly decreased from baseline to third, *Z* = −2.03, *p* = 0.04, from baseline to sixth, *Z* = −2.37, *p* = 0.02, and from baseline to ninth training session, *Z* = −2.20, *p* = 0.02. The NHPT scores also significantly decreased from third to sixth, *Z* = −2.4, *p* = 0.02, and third to ninth training session, *Z* = −2.37, *p* = 0.02. The NHPT decreased 31.5% from baseline (*M* = 49.96 s, SD = 26.85) to ninth training session (*M* = 34.21 s, SD = 7.33).

DextQ-24 scores for subjective experience of dexterity significantly decreased during intervention, $\chi_{\left(3\right)}^{2}=14.92$, *p* < 0.00. The DextQ-24 scores significantly decreased between baseline to sixth, *Z* = −1.28, *p* = 0.01, baseline to ninth, *Z* = −2.54, *p* = 0.01, and third to ninth training session, *Z* = −2.12, *p* = 0.03. The DextQ-24 scores decreased 16.3% from baseline (*M* = 41.4, SD = 15.8) to ninth training session (*M* = 34.6, SD = 16.0).

Jamar dynamometer scores for grip strength significantly increased during intervention, *F*_(3, 21)_ = 5.51, *p* = 0.01. Grip strength (in kilogram) increased 11.3% from baseline (*M* = 23.5 kg, SD = 8.3) to ninth training session (*M* = 26.2 kg, SD = 6.6).

**Figure 5:**
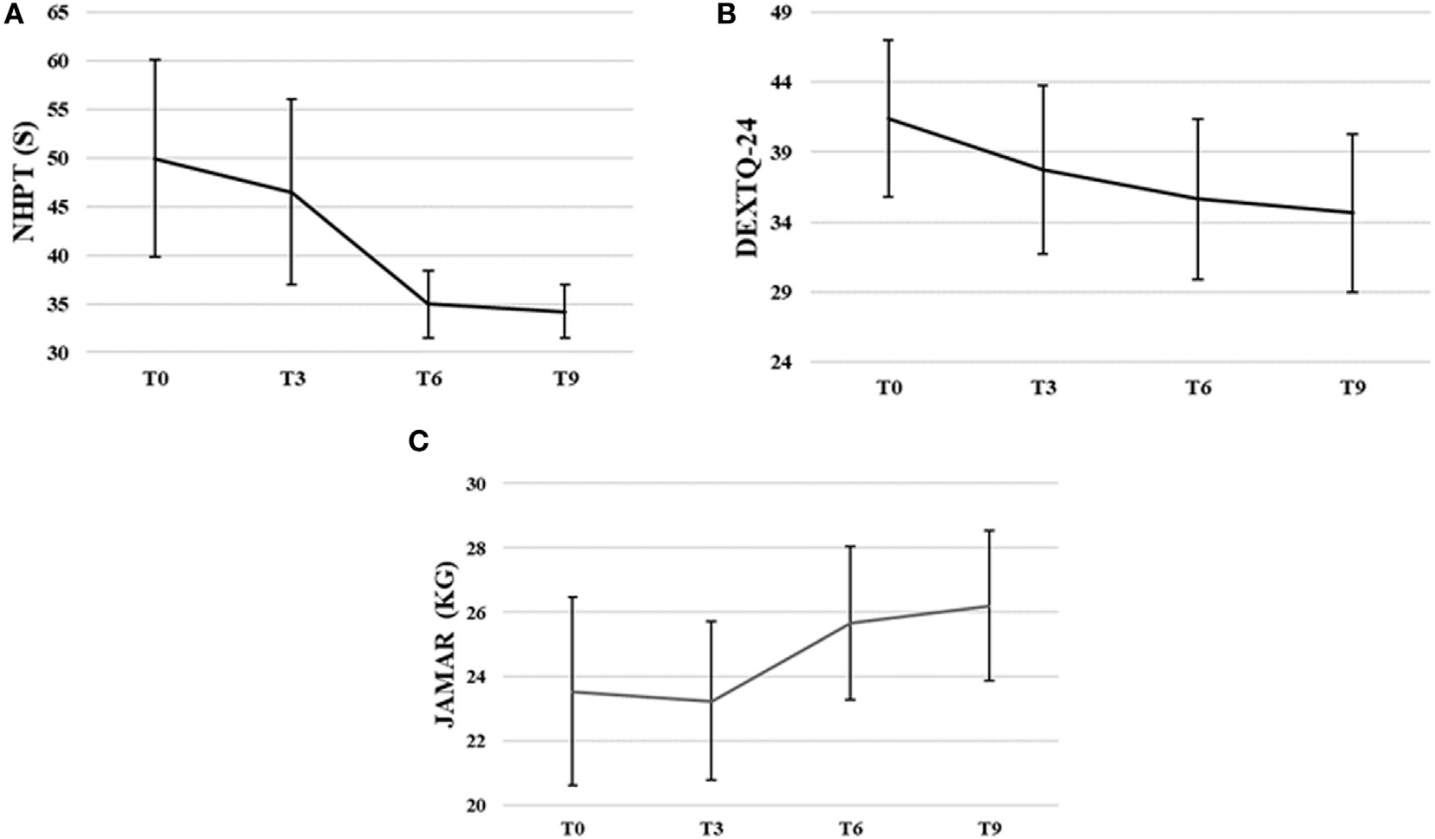
Group means with SEM for the results of **(A)** the Nine Hole Peg Test (NHPT) scores in seconds (s), **(B)** the Dexterity Questionnaire 24 (DextQ-24) scores, and **(C)** the Jamar grip strength in kilograms (kg).

FM-UE scores for motor impairments of the upper limb varied between scores of 42 and 65 at baseline (*M* = 57.5, SD = 9.3), and between 44 and 66 at the ninth training session (*M* = 58.5, SD = 6.7). The FM-UE scores did not significantly change over time (*p* > 0.05) (for more details, see Figures 5A–C).

#### Open End Questions

The first 3 open-end questions revealed that patients had an overall good impression of the therapy and system (7 positive; 1 neutral; 0 negative categorized responses), and that the duration of the therapy (30 min) was fairly good (*N* = 2) to good (*N* = 6). Participants mentioned that the time per session should not be much longer than 30 min, because of loss of concentration and arm-related fatigue. Three participants would consider home-use of the LMC. They stated that it would depend on the price, technical improvements, and the available games. One participant specifically asked to prolong with the VBT and would like to continue at home. The answers on the fourth and fifth question about the possible improvements or overall remarks, revealed that three participants felt that they were either too old for using this kind of technology, were not using a computer at home, or needed help from another person. The majority of participants was enthusiastic about the new experiences with the LMC or had the feeling that they learned from the VBT. Most participants did not have specific practical suggestions to improve the therapy or device, but one participant noticed that it would have been nice to see the personal booked progress in the program of the games. All participants indicated that they benefit from therapist’s additional instructions and were satisfied with the support of a therapist.

## Discussion

The present study aimed to comprehensively explore the usability and feasibility of a 3-week video game-based dexterity training using the LMC in the early rehabilitation phase of stroke patients. We show for the first time that the LMC is a usable tool, measured by the SUS. In addition, stroke patients demonstrated good compliance to the training protocol and were strongly motivated throughout the 3-week training, underlining high feasibility. In addition, hand strength and dexterity significantly improved, both at the level of function and activities/participation.

The reasons why the LMC is usable are multifold. First, LMC is a small, lightweight, USB powered device which can be plugged-in in every computer. Second, the installation of the integrated software is user friendly. Third, no expert technician is needed since there is no need to attach markers of the device to the hands, making the tool beneficial in its use as compared to other VR upper limb tools such as virtual gloves or exoskeletons (45–47). Fourth, the fact the LMC system is relatively cheap, and easy to purchase it may be easily integrated into the home setting. Interestingly, in contrast to Iosa et al. (15), in our sample, also patients with moderately impaired cognitive abilities were included. They also managed to act well with the LMC further underlining its high usability.

### Compliance and Active Participation

Compliance rate was high (87%) and while the only previous LMC study did not report data on compliance rates (15), we consider this high feasibility of VBT with LMC. Active participation level scores were in line with Iosa et al. (15). The slight decrease in PRPS scores after the seventh training session could result from lack of progression in game level difficulty, as all attainable levels were attained after 2 weeks. Such progression in difficulty next to variance and challenge in games is needed to increase the efficacy and compliance. Another reason could be that the LMC is at some point perhaps not sensitive enough anymore to the quality of movement and misses crucial information on upper limb motor recovery (48). For example, the LMC registers the movements as “performed well,” while the real-world movements are still not optimal or contain compensation strategies (49). This could affect the level of challenge in the VBT. Nevertheless, the level of active participation remained very good throughout the training program, but it should be kept in mind that the VBT with LMC was performed in the presence of a therapist during hospitalization and therefore possibly attributing to a high participation level of the patients.

Other studies have examined VBT delivered in the home environment of chronic stroke patients (50, 51). Piron et al. used a tele-rehabilitation approach using two computers, a camera, and a magnetic transmitter/receiver to deliver remote upper limb training in chronic stroke patients with favorable results. All participants completed the intervention, but exact compliance rates were not reported (50). Standen et al. implemented upper limb training with a virtual glove with LEDs and an infrared camera, which proved feasible. However, considerable variation was found both in terms of duration of use (1.46–70% of the recommended duration) and the number of days used (10–100%) (51). In our study, we found high compliance rates and good participation, showing that the LMC system, relatively cheap and simple compared to these systems, is feasible already in the early rehabilitation phase in an in-patient setting. However, home-based implementation of the LMC in the early rehabilitation phase needs further study to determine if compliance rate and active participation would also be acceptable.

From the viewpoint of the therapist, we found that participants were not always completely capable to perform the VBT independently. Visual, verbal, and physical instructions were sometimes needed to support participants. Most therapist instructions were related to depth perception in the VR environment. This is indeed already described previously that stroke patients may experience difficulties in perceiving objects in a 3D environment (52). The gameplay instructions were not embedded in the games and personal progress was not recorded in the software. This prompted the need for a therapist to switch between games, record the personal progress, and intervene when there were technical problems with the LMC. Proposed improvements from patient-reported suggestions are in line with these limitations.

### Efficacy

Although this pilot study was not designed and powered to evaluate efficacy, we found improvements in the objective and subjective dexterity outcomes (31.5% in NHPT, 16.3% in DextQ-24. resp.). This is in line with previous studies (17, 50, 51). The improvements in dexterity may result from the training being intensive, highly repetitive, and task-specific (5). We also found significant improvements (11.3%) in grip strength. There is no resistance of real-world objects involved in the LMC training so this is somewhat unexpected and may be due to spontaneous neurological recovery in the early rehabilitation phase (1, 7, 49). Iosa et al. (15) also found improvements in grasp force after VBT with LMC. Possibly VBT could improve grip strength through the increased number of repetitions of pinch and grip movements but this needs further investigation.

The neurological recovery of motor function of the upper limb was measured with the FM-UE, for which we did not find significant changes. The FM-UE is however a general evaluation of pathological synergies in the upper extremity and not a specific dexterity measure and may fail to detect small improvements in fine manual dexterity and grip strength. In addition, the FM-UE may not be sensitive to the quality of the movement (e.g., if a patient was able to completely perform a movement-item at baseline and performed the same movement-item much smoother at ninth session, it was both scored with a 2, “can be performed”). Furthermore, the baseline scores of our participants were already quite high, leaving less room for improvement.

### Limitations

The present study was subject to limitations, such as the pre-experimental, this means one group pre-test/post-test, design, small sample size, and the limited duration of the intervention. In addition, although the DextQ-24 was validated in patients with Parkinson’s disease (38), it was not formally validated in sub-acute stroke patients.

## Conclusions

The present pilot study is the first to evaluate the usability of the VBT using the LMC to train fine manual dexterity in the early rehabilitation phase of stroke patients as an add-on to conventional therapy. VBT using the commercially available LMC is feasible in the early rehabilitation phase in stroke patients admitted for in-patient rehabilitation. Future studies should investigate the add-on value of home-use of LMC. For home-based training, the software should contain clear build-in instructions for online feedback, options to save and provide feedback on personal progresses and have structured progression and a large variety in challenging games to be successful.

## Ethics Statement

The study was approved by the Ethikkommission Nordwest- und Zentralschweiz of the canton Lucerne. All patients gave informed consent according to the latest declaration of Helsinki (2013).

## Author Contributions

Study design: TV, SF, and EW; data acquisition: TV and SF; data analysis: TV, SF, and EW; interpretation of data: TV, SF, TN, and EW; drafting and revising: TV, SF, TN, and EW; FINAL approval: TV, SF, TN, and EW.

## Conflict of Interest Statement

The authors declare that the research was conducted in the absence of any commercial or financial relationships that could be construed as a potential conflict of interest.

## Abbreviations

CVA, cerebrovascular accident; DextQ-24, Dexterity Questionnaire 24; FM-UE, Fugl-Meyer Upper Extremity scale; LMC, Leap Motion Controller; M, mean; MoCa, Montreal Cognitive Assessment; N, number of participants; NHPT, Nine Hole Peg Test; PRPS, the Pittsburgh Rehabilitation Participation Scale; SUS, System Usability Scale; TT, the total time spent on the intervention; PT, planned time; VBT, videogame-based training.
