# Supplementary material for: Usability of Videogame-Based Dexterity Training in the Early Rehabilitation Phase of Stroke Patients: A Pilot Study
Source: Front Neurol. 2017 Dec 8;8:654. doi: 10.3389/fneur.2017.00654 (PMC5727075; doi:10.3389/fneur.2017.00654)
Supplement: Supplementary file 1 [file data_sheet_1.docx]

**Appendix 1.** Open-end questions

1. Was ist Ihr allgemeiner Eindruck der Therapie mit dem Leap Motion Gerät?

…………………………………………………………………………………………………

…………………………………………………………………………………………………

1. War die Dauer der Therapie für Sie in Ordnung?

…………………………………………………………………………………………………

…………………………………………………………………………………………………

1. Würden Sie das Gerät selber kaufen?

…………………………………………………………………………………………………

…………………………………………………………………………………………………

1. Haben Sie Verbesserungsvorschläge?

…………………………………………………………………………………………………

…………………………………………………………………………………………………

1. Haben Sie allgemeine Tipps oder sonstige Bemerkungen?

…………………………………………………………………………………………………

…………………………………………………………………………………………………

1. War die/der Therapeut/in sachlich und korrekt?

…………………………………………………………………………………………………

…………………………………………………………………………………………………

1. Welches Spiel hat Ihnen am besten gefallen?

…………………………………………………………………………………………………

…………………………………………………………………………………………………

1. Welches Spiel fanden Sie am schlechtesten?

……………………………………………………………………………………………………………………………………………………………………………………
